# Sabolich Prosthetics and Research: A Continuing Journey on the Road Less Traveled

**DOI:** 10.33137/cpoj.v4i2.37089

**Published:** 2021-09-21

**Authors:** S. Sabolich

**Affiliations:** Scott Sabolich Prosthetics and Research, 10201 Broadway Ext, Oklahoma City, USA.

**Keywords:** Health Economics, Orthotics, Prosthetics, Research and Development, Rehabilitation

## Abstract

^**^The Sabolich family had made a unique contribution to both the clinical practice of Prosthetics and Orthotics as well as the business of prosthetics and orthotics. In preparing for this Special Edition, as Co-Editor-in-Chief, I felt it important to include the story of the Sabolich vision for prosthetic clinical practice and their experiences in achieving that vision. Their story is an important part of the history of prosthetic clinical practice in the United States. Their rethinking of what a clinical practice could look like and function as was far-sighted. Achieving it required both risk taking, as well as a new business model. The reaction from other Prosthetic and Orthotic practitioners was strong. Some saw the new model as visionary while others found it provocative. The model was based on a concierge style practice, which provided a premium service to a patient from first appointment to providing and maintaining of their device. They then went further than that by incorporating in-house research into their facility to develop new and innovative prosthetic approaches that would make them leaders in the field of socket design and fit. Their vision was to be a Center of Excellence for Prosthetics and Orthotics Practices, a vison that many people within the sector would likely agree was achieved. I invited current CEO of Scott Sabolich Prosthetics and Research to reflect back on the history of his family's business and how multiple generations of his family were able to see gaps and create opportunities where others could not see beyond the status quo and even fought hard to maintain the status quo. The evolution of this company provides insight into the successful development of novel business practices and economics in Prosthetics and Orthotics, while staying true to core service principles. The result was a business model that, in hind sight, had future-proofed the company in the face of the global economic challenges that impacted the sector in 2008–9 and beyond. It is also an indicator of what opportunities there are, at the business, clinical and technical levels, for those who take the risk of breaking from the status quo. Because of time and capacity constraints on the part of Mr. Scott Sabolich, it was agreed that his professional opinions and insights would be done in an interview format.

## HEALTH HISTORICAL CONTEXT

In the history of Prosthetics and Orthotics in North America, your father, John Sabolich, a second-generation Prosthetist, is remembered for opening a unique Prosthetics and Orthotics Clinic in Oklahoma City that had a patient care model that was completely different from the status quo within the prosthetics sector at that time.

### What do you remember from that time?

My grandfather, Lester Sabolich, opened the Sabolich facility originally in 1947. My dad simply took over the business from his father in the mid eighties. However, it was in the early 90s that dad really put us on the map for O&P.

I remember my father's facilities as being not just a “shop” but more like a Center of Excellence. My father recognized the actual experience of coming to our facilities to receive care should be just as polished and thoughtful as the care itself. If insurance was going to pay for an amputee to get a leg and an amputee could go anywhere in the US, he believed amputees would prefer to go somewhere where an expert team would ensure every moment of that process was personalized, welcoming and thoughtful – and, subsequent history showed he was right.

This has and always will be the guiding light of our business. When my father sold to NovaCare back in 1993, he had eight well-known facilities nationwide. When NovaCare sold to Hanger in 1999, we decided to break off and reinstitute the Sabolich name in Orthotics and Prosthetics (O&P) as Scott Sabolich Prosthetics & Research. It was important that our new clinic mirror the reputation of my father's business and be known as a Center of Excellence for prosthetic care.

Scott Sabolich Prosthetics & Research strives to deliver an unrivaled patient experience from initial contact to delivery. Learning from masters in Customer Service like the Four Seasons Hotels and Resorts or The Cooper Clinic (Toronto), we built destination facilities with the vision that once patients arrived, they were made to feel special from day one through to the end of their program.

### What drove your father to come up with and to invest in this new approach?

At the time, no P&O Clinics were marketing their services on a national level like he was. In addition, he was instrumental in carrying out the research that developed new socket designs for the above knee (AK) amputees, along with Tom Guth CPO and Alan Finnestan CPO. These three changed the way “proper fit” was achieved not only at the AK amputee level but also the below knee (BK), hip, and arm. He recognized that he was doing something very different and new to the industry, and, doubled-down on marketing, invention, collaboration, partnerships, research and development, which was not something that was done at the time in P&O. Finally, rather than establishing several small facilities over the state, he recognized it made more sense to setup a destination facility that could build expertise among a team and nurture the collaboration, research and development that would be needed to push the business forward.

### What did other prosthetists/orthotists say about it?

I was young back then, not getting into the field as a technician until 1989 and attending Northwestern in 1995. What I remember most was that there were two camps of people in our field. One believed my father was a brilliant man and a pioneer in the field and the other who didn't understand his approach and rolled their eyes at every mention of the name Sabolich. I can't help thinking that because he was the first person to recognize the power of marketing in O&P, he may have really provoked some people. But, looking back and looking at our standing in our field, this approach seems to have resulted in our being much more equal in the eyes of other health care workers than how we were seen back in the early nineties.

### What did your patients/clients say about it?

My father assembled teams of very talented prosthetists, technicians, administrative, marketing and research individuals, and most of our patients really appreciated the “Four Seasons like experience” we delivered when getting a prosthesis. This was before the internet and our reputation spread by word of mouth, and did so quickly, by getting exposure on TV shows and national news. This brought people to us from all over the US and other countries.

### What was the underlying business case for Sabolich to get into R&D?

Necessity is the mother of invention and that is what fueled my father and our group. We developed several partnerships, and collaborations to develop enhanced designs for sockets, knees, feet, cosmetics, etc. Without Research AND Development, the O&P field would not be anywhere near where it is today. Fortunately, he and others in the field had the foresight to see the investment in R&D is what would propel our field into the 2000s and beyond.

### What did your father see as his biggest business challenges?

In the 1980s and 1990s, health insurance was still relatively inexpensive and covered most prosthetic and orthotic devices everywhere. My father recognized there would be a tipping point in the world of insurance and there would come a time when coverage would shift, even when it came to putting a price on direct health care and providers.

### Where did he see need for change?

Probably the biggest move he made was to provide the start up capital back in the early 90s for a small non-profit Foundation called Limbs for Life. Limbs for Life, to this day, helps provide sockets and componentry to over 600 amputees nationally every year. These are people with absolutely no insurance coverage for a range of reasons. I am happy to say that I still serve on the board of Limbs for Life and we are well-positioned to experience strong growth, with goals to help twice as many people over the next few years. I am grateful to a group of people, including my father, who put the wheels into motion for this non-profit to flourish, otherwise many lives would be different right now. You can check out what we're doing at limbsforlife.org.

## THE PRESENT AND FUTURE

### Can you describe what your clinical care model today is and how it is different from the ‘typical’ P&O clinical practice?

Scott Sabolich Prosthetics & Research currently has two facilities, one in Oklahoma City and the other in Dallas. We invested heavily in the infrastructure of our clinics and created state-of-the-art facilities not only in the areas visible to patients, but also in the lab where our team spends time honing their craft. Our motto is still the same: “Treat every patient with a “Four Seasons” like experience and act like it's your grandmother you're working on.” Put the patient first, profit a close second, all while not forgetting about your co-workers and how you represent the industry. Our business model focuses on delivering only prosthetic care. Some may call this old-fashioned, but it has allowed us to build and cultivate an expertise among the team that people are willing to travel to seek care from and it's been working for us since 1947.

### How did your own view of the future evolve from the foundation your father built?

Over the years, I've branched out to learn and build expertise in fitting elite Paralympic athletes. I'm very proud of the fact that today the team and I are recognized as experts in parasport. I've also evolved the Research and Development side of our business through partnership with Ottobock. Back in my dad's day, the focus was on switching people from hard wooden or acrylic sockets to soft flexible sockets.^1,2^ Today, almost everyone fits those so the industry's expertise in that area has increased. We have tried to excel in that arena by taking on the complex fittings, whether its Paralympic hopefuls, extremely short AKs, or “Difficult to fit” patients. We have developed a team of experts for each category.

### Was there a specific turning point for you with respect to growing your P&O business in your own direction? If yes, what was it?

In 1999, our team carried on the torch as we left Hanger to bring the Sabolich name back, this rebirth adopted the same model my father used. This worked, for a while. It was around 2012, when I opened the Dallas facility that a new vision began to emerge in my mind. At the time, I was conducting clinical research with Ottobock and recognized the potential for a deeper partnership. We formed a strategic clinical partnership in 2014 and, in 2019, Ottobock made an investment in our patient care division. It was clear to me in 2012 that, in order for the industry to evolve, collaboration with industry leaders like Ottobock would increase innovation and spur growth for all of us. Now, I am working with Ottobock as the CEO of Ottobock Patient Care in North America. We are building a network of best-in-class facilities and working together to deliver the type of unrivaled patient care experience my father dreamed of when he started his business.

### What do you think were missed opportunities by P&O business leadership?

I believe that business leaders in the P&O sector never thought they would see consolidation with manufacturers and O&P Clinics in patient care. This is now within people's awareness and understanding and our profession finds itself at another turning point, but in a position in which they can plan for and react to it.

### What do you think the biggest challenge to the financial future of P&O clinics (non-Covid pandemic related)?

O&P business leaders must resist the urge to cut corners as the result of cutting costs. I am sure none of us spend the amount of time with a patient like we did 20 or even 10 years ago. We must fight to preserve the patient experience and remember to treat each person like a family member. The people we treat have been through a lot, including trauma and some have lived their whole life with a mobility challenge. We owe it to them to spend quality time on their fitting and not let the rush of business override our abilities to be the clinicians we sought out to be. One happy patient will eventually bring you one more patient to make happy. One upset patient can cost you 100.

### What do you think the biggest challenge to the financial future of P&O profession (non-Covid pandemic related)?

The Prosthetics and Orthotics field needs to assert and maintain our position as a valuable player on the health care team for a patient. We have to ensure our documentation means something and that we work with our referral sources, not for our referral sources. As long as Medicare's pricing structure continues to greatly influence how we are paid, there will always be a challenge to adjust our service offerings as there are changes to insurance coverage. I feel like at anytime, Centers for Medicare and Medicaid Services (CMS) could simply adjust the (compensation) scales and, potentially, wipe out half of our industry. This is why we need to support our lobbying efforts for any Medicare changes going forward with the American Academy of Orthotists and Prosthetists (AAOP) and the American Orthotics and Prosthetics Association (AOPA). Its not a problem for some, but for all of us. I also hold strong to the theory of moving toward value-based care verses fee for service. I feel that the new tide is certainly bringing us closer to providing quantifiable value, not simply providing the device, therefore we must embrace the capture of clinical outcome measures in order to be prepared for that requirement.

### Has the Covid pandemic sharpened any of these pain points or has it proven to be a catalyst to support change?

It has been both. While it is difficult to manage with Covid during the normal course of business, some things are easier. Streamlined meetings for one and increased communication within facilities and across the US seem to be strengthened with the use of video conferencing, telehealth, etc. Like everything in the O&P field, we react, we adapt, and we grow because of it.

### As a business owner, what to you think the future sustainable business model (could include production methods, leaning) will be?

Looking ahead, it's clear to be successful in the new world we find ourselves in, we have to diversify the patient care experience. This means maintaining a core destination facility model, while also introducing small mobile units and telehealth service offerings. Here in 2021, the needs of the patient are so different from person to person, town to town, state to state. I certainly think a more digital platform will be utilized supported by a digitally driven operating model, with SLS 3D printing taking over much of the archaic lab techniques used in the past. As a part of Ottobock, an innovation leader, this is an area we are rapidly developing.

### What do the professional associations and schools need to do to get people there?

They need to partner with clinics, manufacturers, and digital platforms to understand where the technology is headed and create opportunities for students to immerse themselves in it early. When the next generation of prosthetists and orthotists get out of school with a Masters in O&P, we need them to be actual Digital Masters of O&P.

## CALL TO ACTION

### What is your single most important call to action?

I live by the quote “What would you do if you knew you could not fail?”. I'd ask every reader to consider what that means for them. Our profession is ripe for change and you can play a role in how it evolves, or you can sit back and let it happen – you choose.

### Who has the power to advocate for or operationalize that change?

The person reading this article.

## DECLARATION OF CONFLICTING INTERESTS

Scott Sabolich is the Founder and Owner of Scott Sabolich Prosthetics and Research. Sabolich Prosthetics and Research is a clinical partner of Ottobock.

## SOURCES OF SUPPORT

None.
